# Age of detection of congenital hearing loss in South Africa: A systematic review

**DOI:** 10.4102/jphia.v16i1.777

**Published:** 2025-01-27

**Authors:** Mukovhe Phanguphangu, Khomotjo Kgare, Andrew J. Ross

**Affiliations:** 1 Department of Family Medicine, School of Nursing and Public Health, College of Health Sciences, University of KwaZulu-Natal, Durban, South Africa; 2 Department of Rehabilitative Sciences, Faculty of Health Sciences, University of Fort Hare, East London, South Africa

**Keywords:** age of detection, congenital, early-onset, hearing loss, early hearing detection and intervention, universal newborn hearing screening

## Abstract

**Background:**

Newborn hearing screening is the most important step towards early intervention of congenital or early-onset hearing loss (HL) and has the potential to optimise patient outcomes. Yet, literature on the age of identification of this HL in South Africa (SA) is sparse.

**Aim:**

To determine the age of identification of congenital HL from published studies in SA.

**Setting:**

The study was conducted in SA.

**Methods:**

This was a systematic review conducted during April 2024 – June 2024 across the following databases: PubMed, EBSCO, Web of Science and Scopus. A random effects (RE) model meta-analysis was conducted using STATA version 18. Statistical tests conducted include meta-summary statistics and RE meta-analysis.

**Results:**

Ten studies, with a total of 1501 children, were included in this review. The computed Q-test of homogeneity was 5870.97 and the pooled mean age of identification was 30 months (95% confidence intervals [CI]: 19.3–41.3).

**Conclusion:**

These findings show that congenital or early-onset HL in SA is identified later than the national and international standards, that is, < 1 month of age. These findings are important for future research, priority setting and implementation of strategies to reduce the burden and impact of HL. Future research investigating the barriers to early detection of HL in SA is recommended. Furthermore, the implementation of evidence-based interventions to lower the age of identification of congenital HL to enable its early detection and intervention in this age group is recommended.

**Contribution:**

This study highlights the need to strengthen preventative programmes for paediatric HL in SA.

## Introduction

Hearing loss (HL) is the most common sensory impairment in children, and it affects an estimated 34 million children below the ages of 15 years globally.^1,2^ Hearing loss can be either congenital or acquired.^3^ Congenital HL, which refers to hearing loss that is present at birth, is one of the most prevalent chronic conditions in children and its incidence ranges globally from 0.5 to 6.0 per 1000 live births.^4,5^ In South Africa (SA), more than 6100 babies are born with or acquire early-onset sensorineural HL annually.^6^

Childhood HL is divided into three types, that is, conductive, sensorineural and mixed. Conductive HL occurs when defects, pathologies or blockages in the outer or middle ear hinder the transmission of sound waves to the cochlea.^7^ Sensorineural HL results from pathology or damage to the cochlea or retro-cochlear structures, which impedes the transmission of sound impulses from the inner ear to the brain.^8^ Mixed HL, as the term implies, combines elements of both conductive and sensorineural HL, arising from issues affecting either the outer or middle ear as well as the inner ear or retro-cochlear structures.^9^ Regardless of its nature, hearing loss can have detrimental effects on childhood development, including delays in speech and language development, poor academic achievement, poor socioemotional well-being and mental health issues.^10^ Moreover, the negative impacts of hearing loss are cumulative, resulting in poorer outcomes even later on in life. For instance, children with HL often experience challenges with securing and keeping employment, getting promotions and earning higher salaries and social integration later in life in comparison to children who had normal hearing during childhood.^11,12,13,14,15^

The increasing global burden of HL and its negative impact on the child, family and society underscore the importance of primary and secondary preventative strategies to reduce the burden of HL.^10^ Primary and secondary prevention include timeous identification through hearing screening and interventions through aural (re)habilitation for HL.^10^ To achieve this, the Joint Committee on Infant Hearing (JCIH) has developed international guidelines for early hearing detection and intervention (EHDI), and locally, the Health Professions Council of South Africa (HPCSA) has developed the position statements to facilitate early identification and rehabilitation of congenital or early-onset HL.^16,17^ As per these guidelines, congenital or early-onset HL should be identified by 1 month, diagnosed by 3 months and (re)habilitation started by 6 months of age.^16^ These guidelines are evidence-based indicating that if children with congenital HL receive intervention by 6 months, they can develop their language and reach their communication milestones at par with that of their normal-hearing peers.^18^

While guidelines on EHDI implementation exist, there have been discrepancies in its implementation in low- middle- and high-income countries; in high-income countries such as the United States (US), more than 98% of neonates receive their screening before hospital discharge.^19^ As a result, there has been an increase in children with HL who have been identified early and can therefore achieve normal cognitive ability.^20^ However, in low- and middle-income countries such as SA, most babies do not receive newborn hearing screening because of a lack of resources, a lack of political buy-in and prioritisation of life-threatening illnesses in state facilities.^10^ As a result, most children with HL are identified much later than the stipulated timelines, sometimes as late as 3 years of age and because of parental concerns about the child’s development rather than through formalised hearing screening programmes.^21,22,23^

The age of identification of HL plays a significant role in the subsequent confirmation and intervention of HL and can have a lasting impact on interventions received and patient prognosis.^10,24,25^ For instance, babies who are identified later than the stipulated guidelines and receive intervention may have persistent delays in communication development that last through to adulthood.^10,24^ Despite the JCIH guidelines^16^ and the HPCSA position statements,^17^ the age of identification of congenital HL in children is variable in SA. Therefore, there is a need to synthesise the research evidence reporting on the age of identification of congenital and early-onset HL in South African children and to achieve a countrywide estimate. This research evidence is important for priority and goal setting for research and clinical practice, as well as the development of public health interventions that aim to ensure early identification of HL and reduce the burden of HL in children. Thus, this review will identify and synthesise the available literature on the age of identification of congenital and early-onset HL in SA and discuss the implications of the findings for clinical practice and future research.

## Methods

### Data sources and searches

We conducted a systematic review and meta-analysis to identify and synthesise the age of identification of congenital or early-onset HL in children in SA. We searched through PubMed, EBSCO, Web of Science and Scopus (each from 01 January 2004 to 31 May 2024), to identify studies that reported the age of identification of congenital or early-onset HL in children in SA. Prior to commencing the systematic review, the protocol for this review was prospectively registered with the International Prospective Register of Systematic Reviews (PROSPERO). Methods are reported according to the Preferred Reporting Items for Systematic Reviews and Meta-Analyses (PRISMA) checklist.^26,27^

Our database search, which was updated on 13 June 2024 to ensure that recently published papers were considered before final results, was conducted using the following key terms: (‘age of identification’ or ‘age of detection’ and ‘congenital’ or ‘early-onset’ and ‘hearing loss’ or ‘deafness’ or ‘hearing impairment’ or ‘hard of hearing’ or ‘hearing disability’ and ‘South Africa’). Limiters included language (only studies published in English were considered) and publication period (only studies published after 01 January 2004 were considered as this was when the JCIH published the guideline for EHDI services).

### Eligibility criteria

The inclusion criteria were specified in the protocol in terms of condition, context and population study designs as follows:

- *Condition*: Studies reporting on congenital or early-onset hearing loss, which is HL occurring in the first few months of life.
- *Context*: This review included all the studies conducted and reported within SA in all contexts, that is, primary healthcare, district, regional, tertiary (academic) and central (academic) hospitals and private healthcare facilities. All studies conducted and reporting data in children outside the borders of SA, which was classified as the context for this study, were excluded.
- *Population*: The population for this study included South African children or permanent residents who were identified with HL before turning 6 years.
- *Study design*: All types of quantitative studies, that is, randomised controlled trials, quasi-experimental studies, non-randomised comparative studies (case-control, prospective or retrospective cohort), non-randomised studies without comparison group (e.g., prospective, or retrospective cohort, cross-sectional), were included.
- *Timeline*: Only studies published between 01 January 2004 and 30 May 2024 were included. Studies published before 2004 were excluded as the first guidelines for the provision of early detection of HL were published in 2003 giving health facilities time to plan and implement EHDI in January 2004.
- *Other*: Only studies published in peer-reviewed scientific journals were eligible for inclusion. Non-peer-reviewed publications, discussion papers, dissertations/theses and conference papers were excluded. Only studies published in English were included in the review.

### Selection procedure

M.P. and K.K. conducted the study selection independently. Articles were exported from the databases into Endnote. The Endnote software was used for screening studies and to record decisions. First of all, duplicate articles were removed. Thereafter, studies were screened based on their titles and abstracts regarding the inclusion criteria, while the researchers were blinded to each other’s decisions. The full-text article was retrieved and reviewed if a decision could not be made based on the abstract. Subsequently, the reference lists of the identified publications were checked for additional studies. Disagreements were resolved by discussion and involving the other research team member (A.J.R.). The full-text PDF versions of the articles that met the inclusion criteria were inspected closely to extract the relevant data.

### Data extraction

Microsoft Excel was used for data extraction and management. A data abstraction sheet was developed to extract the following information from each included study: publication (i.e., authors and date of publication, title of publication, province where the study was conducted), methodology (aim, design, sample size included in the final analysis) and outcomes (i.e., mean age of identification of HL, standard deviations [s.d.]). The data were extracted and exported onto the Microsoft Excel spreadsheet by M.P. and K.K. for all the included studies, with cross and double checking of the data to reduce errors in data entry. After all the data were extracted, a meeting was held to discuss the extracted data. Discrepancies were resolved by discussion between the reviewers. A third reviewer (A.J.R.) was consulted for any unresolved discrepancies.

### Determination of study quality and level of evidence

The methodological quality and the level of evidence of the individual studies included in the review were determined using the Johns Hopkins Research Evidence Appraisal Tool (JHREAT).^28^ M.P. and K.K. independently assessed the study quality and level of evidence using the JHREAT. The JHREAT is specific to certain study designs and was designed to assist reviewers in focusing on concepts that are important to determine a study’s internal validity. Reviewers responded with ‘yes’, ’no’ or ‘not applicable’ to each item on the tool. In the end, every ‘yes’ represented 1-point. The reviewers added up the points to determine the total score and decide whether the study should be rated as high, good or low quality.

To prevent bias, the JHREAT does not provide specific parameters to rate the quality as high, good or low, as each study should be assessed on its own. However, we used parameters from a previous systematic review as a guide on how to rate the quality, where a score of 0–7 was rated as low, 8–10 as good and 11–14 as high. The level of evidence was also determined by the JHREAT based on each study’s design. We used the JHREAT tool to determine the hierarchy of evidence with Level 1 being the highest level of evidence including randomised control trials, Level 2 including quasi-experimental studies and Level 3 including non-experimental study designs. The primary reviewer (M.P.) conducted the quality assessment and the determination of the level of evidence. K.K. cross-checked 100% to ensure reliability. There were no disagreements reported in the appraisal of the articles. Because of the limited information on some reported studies, the authors contacted the corresponding authors of those articles that had missing information to share more data.

### Data management and statistics

Data from included studies were compiled in a Microsoft Excel spreadsheet. The statistical tests conducted included meta-summary, random effects (RE) meta-analysis (forest plot) and meta-regression (relationship between study sample size and effect sizes).

#### Descriptive statistics

Descriptive statistics were used to summarise the studies included in this review including the study author, province of origin, study design, sample size and outcomes, that is, mean age of identification with s.d.

#### Meta-analysis

Meta-analyses were performed only to estimate the pooled mean age of identification of congenital and early-onset HL with 95% confidence intervals (CI). Meta-analysis was conducted when included studies were broadly comparable in terms of study design and outcomes. The primary outcome measure was the mean age of identification of congenital and early-onset HL. For continuous data, as different outcome measures were employed across studies, effect sizes (ES) were calculated as mean age of identification of congenital or early-onset HL in children. We used the s.d. of the mean obtained from each study to compute standard errors which were used in the meta-analysis.

To account for the variability in the methodologies from the different studies included in this study, we used random-effect models to perform each meta-analysis. This increased the validity of the results and allowed the generalising the results to other settings in the country. Because the studies that were included in the review were diverse methodologically, heterogeneity in the results was expected. Heterogeneity arises when differences between study results are beyond those attributable to chance alone; potential sources include the study setting, methodology and sample size among others.^29^ Heterogeneity in this review was assessed using Cochran’s Q-statistic and quantified with the *I*^2^ statistic. A *p*-value < 0.05 was considered statistically significant heterogeneity and this was categorised as low, moderate and high when the *I*^2^ values were below 25%, between 25% and 75% and above 75%, respectively. Sources of heterogeneity were further explored using meta-regression according to the study characteristics, that is, sample size.

In the absence of meta-analysis, that is, because of limited information from the studies and where no response was obtained from the primary authors, primary and secondary outcomes were assessed at the individual study level by narrative synthesis.

### Ethical considerations

Ethical approval was obtained from the University of KwaZulu-Natal’s Biomedical Research Ethics Committee (Ref: 0000BREC/6710/2024). As this was a systematic review, informed consent was not obtained from participants.

## Results

**FIGURE 1:**
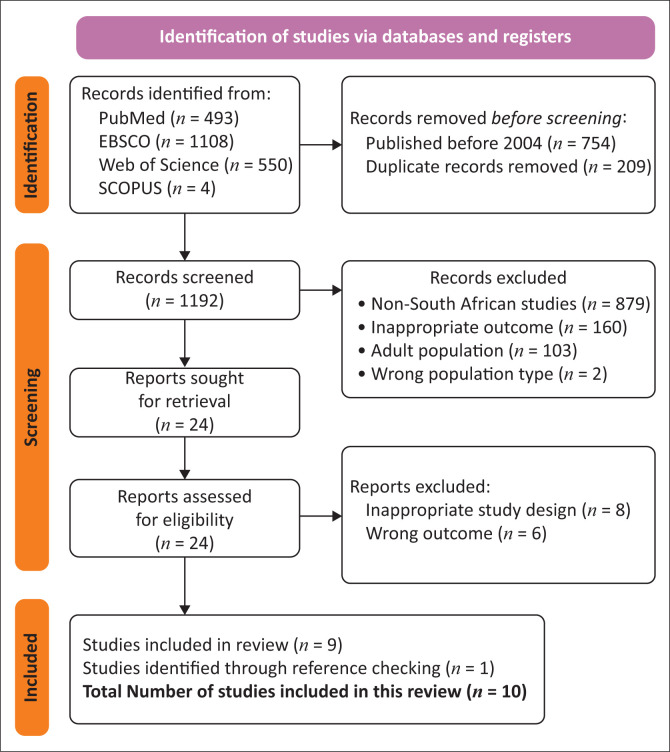
Preferred Reporting Items for Systematic Reviews and Meta-Analyses (PRISMA) flowchart for the study selection process.

We identified 2155 publications from PubMed, EBSCO, Web of Science and Scopus. After removing duplicates and conducting title, abstract and full-text reviews, 10 studies were included in the review as detailed in Figure 1.

### Characteristics of included studies

Table 1 shows the summaries of the 10 studies that are included in this review. Importantly, the Kuschke et al.^22^ study reported data from two data collection sites. Thus, the results from these two data collection sites are reported as Kuschke et al. (A) and Kuschke et al. (B). There was variability in the studies included in this review in terms of the methodologies; eight of the reviewed studies followed a retrospective cross-sectional survey methodology, and two studies followed a quasi-experimental design. The provincial breakdown showed that most studies were conducted in Gauteng (50%, *n* = 5) followed by the Western Cape (30%, *n* = 3). Overall, the total sample included in this study was 1501 children with HL; the smallest study had a sample size of eight participants while the biggest study had a sample size of 315 participants.

**TABLE 1:** Key characteristics of included studies.

Study number	Authors and publication date	Province	Study design	Sample size	Outcomes (mean age in months)	s.d.	Level of evidence	Quality rating
1	Butler et al. (2013)^21^	Free State	Cross-sectional (Retrospective chart review)	260	40.80	-	3	Low
2	Butler et al. (2015)^30^	Free State	Cross-sectional (Retrospective chart review)	48	25.56	-	3	Low
3	Swanepoel et al. (2013)^32^	Gauteng	Cross-sectional (Retrospective chart review)	36	23.70	18.90	3	Good
4	Khoza-Shangase et al. (2014)^31^	Gauteng	Cross-sectional (Retrospective chart review)	70	23.65	-	3	Low
5	Le Roux et al. (2015)^33^	Gauteng	Cross-sectional (Retrospective chart review)	264	11.30	7.80	3	Good
6	Maluleke et al. (2019)^34^	Gauteng	Cross-sectional (Retrospective chart review)	8	27.00	15.41	3	Low
7	Storbeck et al. (2023)^35^	Gauteng	Cross-sectional (Secondary data analysis)	102	28.00	16.50	3	Low
8	Kuschke et al. (2020)^36^	Western Cape	Cross-sectional (Retrospective chart review)	240	22.40	20.60	3	Good
9	Kuschke et al. (2021)^22^	Western Cape	Quasi-experimental	315	48.40	39.00	2	Good
10	Kuschke et al. (2021)^22^	Western Cape	Quasi-experimental	158	52.30	35.10	2	Good

Note: Please see the full reference list of the article Phanguphangu M, Kgare K, Ross AJ. Age of detection of congenital hearing loss in South Africa: A systematic review. J Public Health Africa. 2025;16(1), a777. https://doi.org/10.4102/jphia.v16i1.777, for more information.

s.d., standard deviation.

### Critical appraisal for the quality and level of evidence

All included studies were assessed using the JHREAT.^28^ Study quality ratings and level of evidence are shown in Table 1.

### Age of identification of hearing loss

**FIGURE 2:**
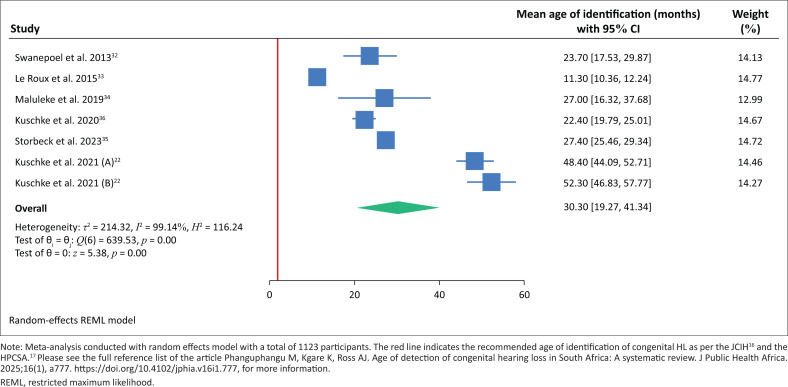
Mean age of identification of congenital or early-onset hearing loss in South Africa.

The mean age of identification of HL ranged from 11.3 to 52.3 months. Three studies (i.e., Butler et al.,^21^ Butler et al.^30^ and Khoza-Shangase et al.^31^), were not included in the meta-analysis because of limited information. Seven studies were included in the meta-analysis conducted using random-effect models as shown in Figure 2.

The pooled mean age of identification of congenital HL was 30 months (95% CI: 19.3 – 41.3). However, the meta-analysis showed high heterogeneity (*I*^2^ = 99.14%, Cochran Q-statistic [639.53], *p* = 0.00) among the studies and as a result, we performed a REs meta-regression to identify sources of this high heterogeneity. Meta-regression results, as shown in Table 2, showed that a positive relationship exists between the meta-effect sizes and the sample size of each study, despite being statistically insignificant (*p* = 0.94) at the 5% level.

**TABLE 2:** Meta-regression of effect sizes and sample sizes (*N* = 7).

_meta_es	Coefficient	s.e.	*t*-statistic	*P* > | *t* |	95% CI
Sample size	0.0030933	0.0416638	0.07	0.941	−0.0785663 to 0.0847528
_cons	29.6438	10.90812	2.72	0.007	8.264283 to 51.02331

Note: Effect size: mean age of identification in months; RE meta-regression; method: REML; residual heterogeneity; tau^*2*^ = 256.9; *I*^*2*^ (%) = 99.15; *H*^*2*^= 117; *R*-squared (%) = 26.55; Wald chi^*2*^(1) = 0.01; Prob > chi^*2*^= 0.94. Test of residual homogeneity: *Q* res = chi2(5) = 581.7 Prob > *Q*_res = 0.0000. Please see the full reference list of the article Phanguphangu M, Kgare K, Ross AJ. Age of detection of congenital hearing loss in South Africa: A systematic review. J Public Health Africa. 2025;16(1), a777. https://doi.org/10.4102/jphia.v16i1.777, for more information.

s.e., standard error; 95% CI, confidence interval; REML, restricted maximum likelihood.

### Narrative synthesis of the three studies not included in the meta-analysis

We contacted the authors of the three studies that had limited information (i.e., Butler et al.,^21^ Butler et al.^30^ and Khoza-Shangase et al.^31^) to provide further information or share their datasets. However, no response was obtained. All three studies were retrospective and conducted in hospital settings. Two were conducted in the Free State while one study was conducted in Gauteng. From these studies, the average age of identification ranged from 25.6 to 40.8 months in the Free State, while in the Gauteng study, it was 23.7 months. While these studies were not included in the meta-analysis, their age of identification fell within the age range from the meta-analysis, that is, 30 months (95% CI: 19.3–41.3).

## Discussion

This is the first systematic review to synthesise data on the age of identification of congenital and early-onset HL in SA. This review found 10 studies reporting the age of identification of congenital HL in SA. Of these, eight were retrospective chart reviews while two studies were quasi-experimental. Although SA has nine provinces, the majority of research was concentrated in Gauteng (*n* = 5 studies), and the Western Cape (*n* = 3 studies), the two most developed provinces in SA. Only two studies were conducted in the Free State province.

Considering the 1 month age of identification emphasised in the national and international standards,^16,17^ findings from this review, that is, pooled mean age of identification of 30 months, showed that SA is still not meeting the recommended standards. These findings also demonstrate a lack of success in meeting the newly set guidelines of 1-2-3 benchmark,^16^ let alone the initial 1-3-6^17,37^ benchmarks that were set for congenital and early-onset HL in SA. These findings highlight the urgent need for improved early detection practices in the South African context^10^ as well as the need to intensify efforts towards realising EHDI benchmarks^16^ in the country.^10^ Other low- and middle-income countries such as India and Brazil have reported similar findings as those found in this study. For instance, a recent study in India reported the mean age of identification of HL as 18.6 months (s.d.: 12.3) while a Brazilian study reported the mean age of 24 months (s.d.: 27.48).^38,39^

Despite the JCIH publishing the EHDI guidelines in 2004 and advocating for their implementation, research evidence shows that most healthcare facilities in SA still do not provide universal newborn hearing screening.^10,34,40,41,42,43,44,45^ This lack of universal newborn hearing screening in most healthcare facilities could be a contributing factor to the late detection of congenital and early-onset HL in SA. A range of systemic challenges that negatively affect the provision of EHDI services in SA have been reported in previous studies.^10,24,41,42,43,44,46,47,48^ These include a shortage of audiologists to conduct the screenings, limited funding necessary to procure screening equipment, inadequate prioritisation of newborn hearing screening services and deficient maternal awareness on the importance and access to newborn hearing screening services.^10,24,41,42,43,44,46,47,48^ For instance, a recent survey in the Eastern Cape province showed that only 50% of public hospitals (8 out of 16) offered newborn hearing screening; however, among these, only 13% (*n* = 1/8) provided universal newborn hearing screening while the remainder implemented only risk-based screening practices.^10^ Similar trends have been observed in KwaZulu-Natal and Gauteng provinces, where studies indicate that many hospitals do not provide newborn hearing screening because of insufficient audiologists, lack of screening equipment and the early discharge of infants without complications – often before screenings can be administered.^41,42,43,44,46^ Unfortunately, reliance on risk-based screening often results in many infants without identified risk factors being overlooked until later stages, at which point any interventions may yield diminished effectiveness in relation to speech and language development.^10,49^

To address the absence of universal newborn hearing screening in hospitals and to realise the 1-3-6 benchmark, the HPCSA updated the EHDI guidelines to incorporate alternative screening contexts, such as primary healthcare clinics and community initiatives led by community health workers (CHWs).^17^ Two studies conducted in the City of Cape Town have demonstrated that infants and newborns can be identified before reaching 1 month of age, aligning with the 1-3-6 benchmark and facilitating the early diagnosis and subsequent intervention of HL.^50,51^ This is aligned with SA’s primary healthcare objectives, that is, health promotion and disease prevention, especially given that primary healthcare serves as the initial point of contact within the healthcare system.^52,53,54,55,56^ However, despite the efficacy shown by the two studies in Cape in achieving the 1-3-6 benchmark, to date there still remains a lack of widespread implementation of these initiatives. Consequently, many South African children are still without access to these EHDI services.^10,40,43,46^

Findings from this review highlight the need to develop context-specific practices that aim to reduce the age of identification of HL,^38,39,57^ which is particularly important when given SA’s ongoing healthcare reforms aimed at implementing Universal Health Coverage through National Health Insurance.^58,59^ To facilitate the early detection of congenital HL, there exists an opportunity to scale up community-based screening by integrating newborn hearing screening into immunisation clinics.^60^ This approach could ensure that all babies either born in or out of hospital receive hearing screening services. This accounts for those who are discharged from the hospitals prior to undergoing hearing screening,^10^ as well as those born outside of hospital settings, that is, those born before arrival, those born at maternal obstetric clinics, community health centres or at home.^61^ The added advantage of using immunisation clinics is the fact that over 90% of infants in SA participate in immunisation programmes within their first year of life because of the Expanded Programme on Immunisations.^62^

In this instance, primary healthcare nurses and CHWs could be trained to conduct screenings.^51,63,64^ Previous studies have shown that nurses are overburdened and incorporating newborn hearing screening into their scope would increase their workload.^41,43,46^ However, initial findings from an ongoing study in KwaZulu-Natal have found that nurses were keen to conduct the screening, provided they are provided with screening equipment and also receive the necessary training. In addition to nurse-administered screening, global trends are shifting towards the training of CHWs in providing mid-level health work such as hearing screening and as a result, CHWs could be considered for conducting this screening.^63,64^ Moreover, further pilot studies should explore a task-shifting interdisciplinary approach wherein newborn hearing screenings are offered concurrently with vaccinations during immunisation visits, ensuring timely identification of HL. This is important for a country like SA where travel time and transport costs have been found to have a negative impact on access to health services.^65^ Thus, offering the two services concurrently would ensure that parents do not have to return to clinics for hearing screening only. In relation to meeting the 1-2-3 or the 1-3-6 guidelines, offering the services concurrently would ensure that subsequent services to hearing screening such as confirmation and management of HL are offered timeously.This is especially crucial, considering that evidence suggests that when aural rehabilitation is provided within the first 6 months of life, infants with HL are able to develop communication and cognitive skills at par with their normally hearing counterparts.^66,67,68,69^

We have identified two strengths of our study. Firstly, the extent of the systematic search, which includes four different databases, and extensive use of reference lists to identify studies. Secondly, to the best of our knowledge, this is the first systematic review with meta-analysis to synthesise the age of identification of congenital and early-onset HL in SA, which utilised random-effect models and meta-regression to account for heterogeneity. Nevertheless, this review has limitations to consider. Firstly, although the final search yielded 10 studies, three studies were not included in the meta-analysis because of inadequate information. Thus, the age of identification from our meta-analysis could over or underestimate the true age. Secondly, our data came from studies with different methodologies, that is, retrospective, cross-sectional, prospective cohort and quasi-experimental studies, leading to high heterogeneity and made conducting the meta-analysis challenging. However, we conducted our meta-analysis using a random-effect model, which accounted for the observed variability and high heterogeneity, increasing the validity and the generalisability of the data to different contexts in the country. Furthermore, we conducted a subgroup analysis, to account for variability in the study settings and methodologies used to determine the age of identification of congenital or early-onset HL.

## Conclusion

Early identification of HL in children is crucial to the follow-up services such as confirmation and management of HL. The aforementioned services are important in ensuring that the identified HL is addressed timeously to ensure that the child develops to be at par with normal hearing peers. However, in the current study, an age at identification of 30 months was determined. This indicated that congenital or early-onset HL in SA is identified later than the local and international best practice guidelines, that is, < 1 month of age. These findings highlight the need to strengthen health systems and to develop context-specific programmes that can increase access to early identification of congenital HL in SA.

Based on these findings, we recommend the following:

- Public health awareness and policy reviews to escalate the priority placed on EHDI programmes and to mandate EHDI to be part of the Maternal and Child Health package of care rather than stand-alone programmes.
- Implementation trials that explore the other models of early identification of congenital HL in children in SA such as the scale up of community-based and primary healthcare-based screening programmes.
- Further research aimed at formalising and standardising and ensuring the sustainable provision of newborn hearing screening, especially at primary healthcare where majority of babies receive their immunisations.
- Health systems strengthening through collaborative and task-shifting practices that enhance and add on to hospital-based screenings such as community-based and primary healthcare-based screenings.
